# Dopamine Induces Optical Changes in the Cichlid Fish Lens

**DOI:** 10.1371/journal.pone.0010402

**Published:** 2010-04-29

**Authors:** J. Marcus Schartau, Ronald H. H. Kröger, Bodil Sjögreen

**Affiliations:** Department of Biology, Lund University, Lund, Sweden; University of Lethbridge, Canada

## Abstract

The crystalline lens in the cichlid fish *Aequidens pulcher* undergoes a transformation of its optical properties every dawn and dusk as the eye adapts to changes in light conditions. During dusk the transformation result in an increase of the refractive power in the lens cortex, the outermost 40 percent. The change is thought to match the optical properties of the lens to the requirements of the retina. Using a short term *in vitro* lens culturing system together with optical measurements we here present data that confirm that the optical properties of the lens can change within hours and that dopamine influences the optical properties of the lens. Dopamine yields dose-dependent decrease of the refractive power in the lens cortex. The D1-agonist SKF-38393 induces a similar decrease of the refractive power in the cortex, while the D2-agonist quinpirole has no effect. The effect of dopamine can be blocked by using the D1-antagonist SCH 23390. Our results suggest that dopamine alone could be responsible for the light/dark adaptive optical changes in the lens, but the involvement of other signaling substances cannot be ruled out.

## Introduction

Vertebrate eyes share a common ancestry and the general design appears to have been preserved over the past 500 million years. Similarities between vertebrate groups are present in both general morphology and development of the retina and lens as well as on a cellular level, for instance the photoreceptors and their opsins [1]. A shared trait through the entire vertebrate linage is the multifocal optical system that compensates for chromatic aberration. These systems are present in all major vertebrate groups, from lampreys to primates [2], [3], [4], [5], [6].

Chromatic aberration arises from the same principle by which a prism divides white light into its spectral components: refraction is wavelength-dependent where longer wavelengths are refracted less than shorter. The defocusing effect of chromatic aberration is most severe in powerful lenses with large apertures, because such lenses have short depth of focus. Although multifocal optical systems occur throughout the vertebrate linage, it is best understood in teleosts (bony fishes) where the crystalline lens is the sole refractive element [7]. To achieve multifocality, teleost lenses have slight variations in their refractive index profiles, which divide the lens into concentric refractive zones, each having a different focal length in monochromatic light and focusing a different wavelength, or color, onto the retina in polychromatic light. The wavelengths in focus at the retina match the photoreceptor's maximum sensitivities, their λ_max_. This creates a well-focused image on the retina composed of relevant wavelengths despite chromatic aberration [6].

In most teleosts retinomotor movements result in a dramatic change in the outer layers of the retina during light/dark adaptation. The process is composed of two parts; a shift in position between the cone and rod inner/outer segments, and a migration of melanin granules in the retinal pigment epithelium. Circadian changes in the retina are controlled not only by light intensity variations, but also by endogenous signaling substances. Some changes are specifically activated by fluctuations in dopamine level, which acts as a signal for both light-induced and circadian endogenous light adaptation of the eye. Ocular dopamine is produced solely by one type of interplexiform cells in the retina from where it diffuses freely into the vitreous [8], [9]. During day the free concentration of dopamine in the vitreous is three times higher than during night [10], [11]. Dopamine directly influences both parts of the retinomotor movements by triggering pigment dispersion in the retina pigment epithelium and by inducing contraction of the cone myoid which pulls the cone inner/outer segment into the assumed focal plane while the rod inner/outer segments are pushed to a more distal position [12]. This gives the animals a retina that is pure cone during day and pure rod during night. As a trichromate, A*equidens pulcher*, a South American cichlid, has its three spectral cone types in the focal plane during day that are replaced at dusk by one set of rods [13], [14], [15].

The lens optical properties change as the eye dark-adapts to compensate for the change occurring in the retina during dusk, where the animals' wavelength discrimination ability (color vision) disappears and λ_max_ changes. The optical properties of the lens change from multifocal during day towards monofocal during night, which is thought to compensate for the change occurring in the retina from three active photopigments to one. The change in the lens results in an increase of the refractive power in the outermost 40 percent of the lens radius. A similar, but stronger increase of the refractive power in this region occurs if the eye is depleted of dopamine, which suggests that dopamine is directly or indirectly involved in the regulation [16].

We used an *in vitro* short-term culturing technique and treated the lenses with dopamine and dopamine analogs in order to determine whether dopamine is directly responsible for the changes occurring during light-dark adaptation and dopamine depletion. We found that dopamine influenced the lens directly and decreased the refractive power in the outermost 40 percent of the lens. Furthermore, we gained insight into what mechanism regulates the changes in refractive power by determining that the D1 receptor family is directly involved in the regulation while the addition of D2 family agonists had no effect.

## Results

**Figure 1:**
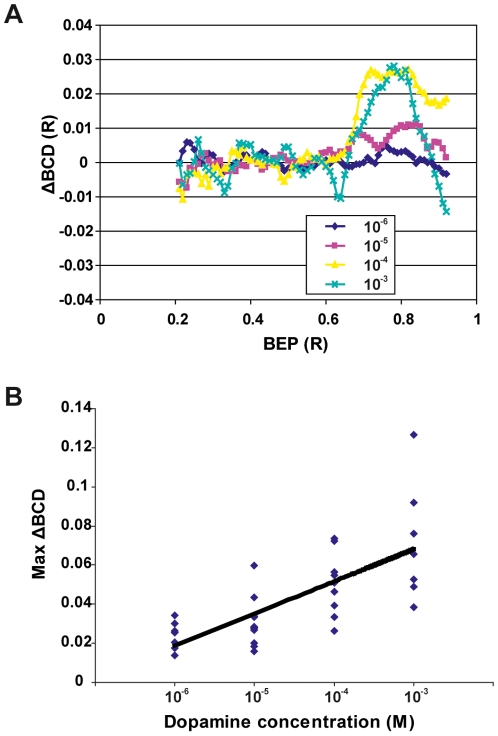
Dopamine decreases the refractive power in the lens periphery. (A) The mean ΔBCD curves describe the difference between the treated and untreated lenses from all animals (see Methods and Materials). Positive ΔBCD values indicate a decrease in refractive power *i.e.* an increase in BCD in the treated lens compared with the corresponding control lens from the same animal. The curves are truncated at 0.2 lens radius (R) and 0.95 R because the scanning method has low accuracy close to the optical axis and in the outer periphery of the lens [30]. These regions contribute little to the image because of a small effective aperture (central region) and reflection at the lens surface (peripheral region; [31]). Note from the mean ΔBCD curves that dopamine decreased the refractive power in the lens cortex, from 0.60 R and outward. The mean ΔBCD increased in BEP 0.60–0.95 R when treated with increasing concentrations of dopamine. The curves represent the mean ΔBCDs from 34 animals (10^−6^: n = 7, 10^−5^: n = 11, 10^−4^: n = 9, 10^−3^: n = 7). (B) Linear regression on max-ΔBCD as a function of dopamine concentration gave a slope with the r^2^ value 0.457 that differed significantly from 0. Max-ΔBCD is the largest difference in BCD between the control lens and the treated lens measured at BEP 0.55–0.95 R (n = 34).

Dopamine decreases the refractive power in the lens cortex, *i.e.* the outermost 40 percent of the lens radius. Treating lenses *in vitro* with dopamine, at concentrations from 10^−6^ to 10^−3^ M, yielded a dose-dependent response (figure 1A). The change in refractive power is described as ΔBCD. BCD, or back center distance, is the longitudinal distance in a meridional plane from the lens center to where a laser beam deflected by the lens intercepts the optical axis. BCD is a function of BEP, or beam entrance position, *i.e.* the lateral distance between the entering laser beam and the optical axis of the lens. ΔBCD is the difference in BCD between a treated and an untreated lens from the same animal. Positive ΔBCD values indicate that the treatment decreased the refractive power while negative values describe the opposite. By comparing the variation in the ΔBCD/BEP curves the maximum variation in the ΔBCD, _max_ ΔBCD, was determined for the lens pair from each animal. A linear regression performed on the _max_ ΔBCDs yielded an r^2^ value of 0.457 and a slope that was significantly different from 0 (p<0.001) (figure 1B).

**Figure 2:**
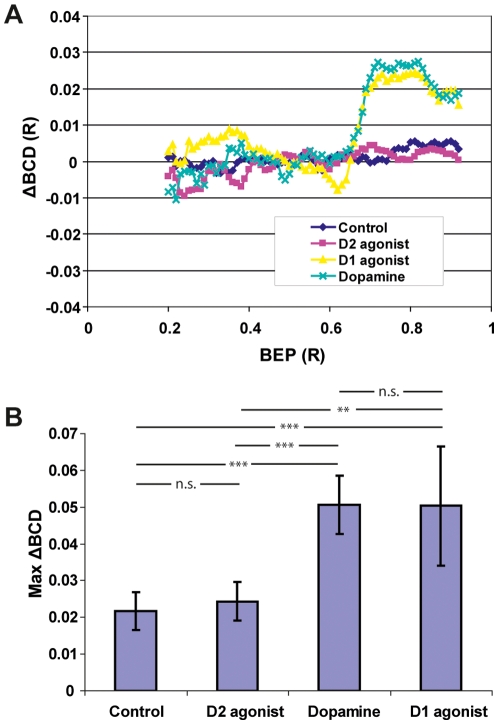
Dopamine and a D1-agonist induce similar changes in lens refractive power. (A) Mean ΔBCD curves from 31 lens pairs where one lens was treated with dopamine, D1-agonist, or D2-agonist, as well as pairs where both lenses were untreated (control). Treatment with the D1-agonist SKF-38393 induced an effect similar to that of dopamine in that BCD values increased in the lens cortex, BEP 0.60–0.95. Both treatments thus reduced the refractive power in the cortex compared to untreated lenses. The D2-agonist quinpirole had no effect. The curves represent the mean ΔBCDs from 31 animals (Control: n = 8, D1: n = 7, D2: n = 7, D: n = 9). (B) Max-ΔBCD values and standard deviation in the control and treatment groups. There was no significant (n.s) difference between max-BCD values from dopamine and D1-agonist treatments or between the control and D2-agonist. ** = Significance level 0.01%, *** = significance level 0.001% (n = 31).

Experimental groups where either dopamine, D1- or D2-agonists were used was compared to a control group where both lenses remained untreated. ΔBCD curves from experiments with 10^−4^ M of the D1-agonist SKF-38393 were similar to those where dopamine was used. In contrast, ΔBCD curves from experiments with 10^−4^ M of the D2-agonist quinpirole were similar to curves from the control group (figure 2A). Statistical analysis with permutational analysis of variance (PERMANOVA) shows that dopamine and the D1-agonist induced changes in the refractive power in the same direction. The differences from the control were statistically significant for both treatments (p<0.001), while treatment with the D2-agonist did not induce any significant difference from the control (p = 0.496). The dopamine treated group did not differ from the group treated with D1-agonist (p = 0.971) (figure 2B).

**Figure 3:**
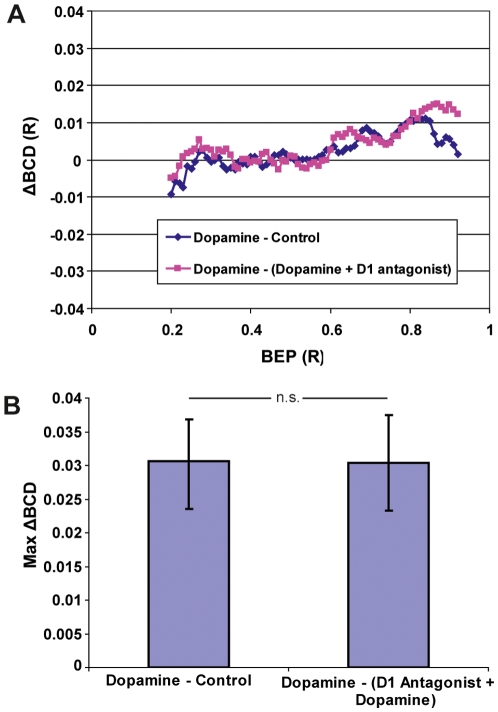
The effect of dopamine is abolished by a D1-antagonist. (A) ΔBCD curves from 11 lens pairs where one lens was treated with dopamine and the other lens remained untreated are compared with ΔBCD curves from 10 lens pairs where one lens was treated with dopamine and the other lens with dopamine and the D1-antagonist SCH 23390. The curves are similar, indicating that the D1-antagonist abolished the effect of dopamine in the lens. The curves represent the mean ΔBCDs from 21 animals (Control: n = 11, D1 antagonist: n = 10). (B) The max-ΔBCDs and standard deviation. Both groups were similar with no statistically significant difference between them. This indicates that the D1-antagonist abolished the effect of dopamine. There was no significant (n.s) difference between the two groups (n = 21).

ΔBCD curves obtained when one lens was treated with dopamine and the other remained untreated were compared to ΔBCD curves obtained when one lens was treated with dopamine and the other treated with dopamine and the D1-antagonist SCH-23390. The ΔBCD curves are similar and there was no statistically significant difference between the groups (p = 0,431) (figure 3).

## Discussion

During light adaptation the refractive power of the lens cortex, the outermost 40 percent, is reduced. Dopamine levels are roughly three times higher in the vitreous during day than night and if dopamine is directly controlling the lens' refractive power, treatment with dopamine should induce a reduction of the refractive power in the same layers. The results obtained in this study are consistent with the hypothesized scenario; treatment of lenses *in vitro* with dopamine leads to a decrease in refractive power in the outermost 40 percent of the lens cell layers. The reduction in refractive power increases with increased dopamine concentration in a dose-dependent manner within the concentration range tested.

The D1-receptor family, consisting of the D1_A1_, D1_A2_, D1B, and D1C receptors in teleosts [17], [18], appears to be involved in the observed regulation since the D1-agonist induced a similar response as dopamine, while the D2-agonist had no effect even at the increased concentration used. A D1-antagonist was used in conjunction with dopamine in order to test whether dopamine and the D1-agonist were actually binding to a receptor. The D1-antagonist abolished the effect of dopamine, which supports the conclusion that a D1-receptor is involved. It is currently unknown where these D1-receptors are located and what specific cellular mechanism they activate. Two plausible effects of D1 family receptor activation are increased production of cAMP and phosphoinositide hydrolysis. All of the four known teleost D1 receptor subtypes increase cellular levels of cAMP [18]. cAMP moves freely through certain gap junctions [19], [20], which in turn means that the receptors could either be localized in the epithelial layer, or spread out through the affected cell layers. In the retina during light-adaptation, dopamine modulates the gap junctions in the horizontal and amacrine cells through cAMP. The modulation is most likely regulated through phosphorylation of gap junction proteins by the cAMP-dependent protein kinase, PKA [21]. In the sheep lens, PKA phosphorylates gap junction proteins in the lens cortex but not the lens core [22]. It is possible that dopamine through phosphorylation of the cortex gap junction proteins can modulate the lens' internal fluid circulation, which is responsible for maintaining homeostasis and distributing nutrients in the lens [23]. Furthermore, the circulation is strongest in the lens cortex where dopamine has an optical effect [24]. Phosphoinositide hydrolysis results in two separate signaling pathways; increased intracellular calcium levels through inositol trisphosphate, IP_3_, and increased activation of protein kinase C, PKC, through diacylglycerol. In mammalian lenses, PKC appears to have gap junction regulatory properties in the epithelial cells and could possibly therefore also affect the lens circulation [25], [26]. Calcium stores have been described in the epithelial layer of the sheep lens [27] but not to our knowledge in teleost lenses. Ca^2+^ by itself influences the water permeability of the aquaporin AQP0 in the lens cortex, which in turn might affect the lens circulation [28]. Regardless of the initial effect induced by dopamine, a modulation of the lens circulation seems the most plausible explanation for the optical regulation in the lens observed in this study.

Our results are consistent with that dopamine alone may be responsible for the optical change in the lens occurring between night and day. If so, the lens would be in its natural state during night and the increased dopamine levels during day would decrease the refractive power in the lens periphery until dusk when the dopamine levels drop again. In the retina, however, several compounds work together and form a push-pull system that transforms the retina during dusk and dawn [12]. Further investigations are required to determine what other mediators, if any, are involved in lenticular light-dark adaptation and how this signal propagates from cell to cell.

### Conclusion

Dopamine directly induces a decrease in refractive power in the outermost 40 percent of the lens cell layers and acts through a D1 receptor family regulated pathway. The change is similar to that occurring between night and day.

## Materials and Methods

### Ethics Statement

All experiments involving animals were approved by the regional ethical committee for animal research, Malmö/Lunds djurförsöksetiska nämnd.

### Animals

Fishes, *Aequidens pulcher*, where obtained through a local distributor and kept in aquaria under 12h-12h light-dark cycles. The animals used in the experiments were isolated 24h prior to the experiments by translucent dividers in their aquaria to avoid damage to the eyes and effects caused by stress and aggression. All experiments were initiated 3 hours after subjective daybreak to exclude circadian influences.

### Incubation and optical measurements

The animals were killed through pithing and the lenses were excised immediately. The lenses where cultured for four hours at room temperature, 20–22°C, in a modified H10 medium, 120 mM NaCl, 2.50 mM KCl, 0.80 mM CaCl_2_, 1 mM MgCl_2_, 10 mM glucose and 3 mM HEPES. The pH was adjusted to 7.3 and osmolarity set to match that of the vitreous (330 mOsm) by using a slightly more concentrated solution.

During culturing, both lenses remained untreated in the control group. In the experimental groups, one lens from each animal was treated, while the other lens remained untreated as internal control. Right and left lens was alternated as control. Lenses were treated with either dopamine hydrochloride, the D2 agonist (±)-quinpirole dihydrochloride, the D1-agonist (±)-SKF-38393 hydrochloride, or dopamine hydrochloride in combination with the D1-antagoinst (+)-SCH-23390 hydrochloride. All substances were obtained from Sigma Aldrich and dissolved in culturing medium. All substance concentrations were obtained from the literature. The concentration of the D2-antagonist was increased due to lack of response at the initial concentration [29].

The lenses' optical properties were determined with laser scanning that measures the back center distance (BCD) at each beam entrance position (BEP). BEP is the lateral distance from the optical axis to where the laser beam enters the lens and BCD is the longitudinal distance from the center of the lens to the where the beam deflected by the lens intercepts the optical axis. BCD as a function of BEP gives a longitudinal spherical aberration (LSA) curve representing the variation in refractive power over the lens radius. Since the lenses are spherically symmetric, the results from both halves of the lens diameter were averaged, which removes artifacts that occur because of small errors in the position of the optical axis. More in-depth descriptions have been published previously [30].

### Data processing and statistics

To reduce the impact of individual variation, the LSA curve from the untreated lens (control) was subtracted from the LSA curve from the treated lens in each animal. This yielded a ΔBCD curve that describes the difference in BCD between right and left lens. Untreated lenses from the same animal had very similar LSA curves and any deviation from 0 in the ΔBCD curve therefore indicated the effect of the treatment. For further comparisons the maximum deviation, max ΔBCD, was measured in each ΔBCD curve. The max ΔBCDs from the different treatments were used in statistical analyses. First, linear regression was used on the max ΔBCD from the dopamine concentration series and the statistical significance between the slope and 0 was tested with ANOVA (n = 34). Second, permutational ANOVA (Primer v6.0) was used to test for any statistical difference in max ΔBCD between agonists and control (n = 31). Finally, permutational ANOVA was also used to compare max ΔBCD from lenses treated either with dopamine and antagonist or only dopamine (n = 21).

## References

[1] Lamb TD, Pugh EN, Collin SP (2008). The origin of the vertebrate eye.. Evo Edu Outreach.

[2] Gustafsson OS, Collin SP, Kröger RHH (2008). Early evolution of multifocal optics for well-focused colour vision in vertebrates.. J Exp Biol.

[3] Malmström T, Kröger RHH (2006). Pupil shapes and lens optics in the eyes of terrestrial vertebrates.. J Exp Biol.

[4] Lind OE, Kelber A, Kröger RHH (2008). Multifocal optical systems and pupil dynamics in birds.. J Exp Biol.

[5] Karpestam B, Gustafsson J, Shashar N, Katzir G, Kroger RH (2007). Multifocal lenses in coral reef fishes.. J Exp Biol.

[6] Kröger RHH, Campbell MC, Fernald RD, Wagner HJ (1999). Multifocal lenses compensate for chromatic defocus in vertebrate eyes.. J Comp Physiol [A].

[7] Matthiessen L (1886). Über den physikalisch-optischen Bau des Auges der Cetaceen und der Fische.. Pfluegers Arch.

[8] Ohngemach S, Hagel G, Schaeffel F (1997). Concentrations of biogenic amines in fundal layers in chickens with normal visual experience, deprivation, and after reserpine application.. Vis Neurosci.

[9] Witkovsky P, Nicholson C, Rice ME, Bohmaker K, Meller E (1993). Extracellular dopamine concentration in the retina of the clawed frog, Xenopus laevis.. Proc Natl Acad Sci U S A.

[10] Dearry A, Burnside B (1989). Light-induced dopamine release from teleost retinas acts as a light-adaptive signal to the retinal pigment epithelium.. J Neurochem.

[11] Delgado MJ, Cespedes MV, De Pedro N, Alonso-Bedate M, Alonso-Gomez AL (2001). Day/night variations of dopamine ocular content during *Xenopus laevis* ontogeny.. Neurosci Lett.

[12] Burnside B (2001). Light and circadian regulation of retinomotor movement.. Prog Brain Res.

[13] Burnside B, Nagle B (1983). Retinomotor movements of photoreceptors and retinal pigment epithelium: mechanisms and regulation.. Prog Retin Res.

[14] Kolbinger W, Kohler K, Oetting H, Weiler R (1990). Endogenous dopamine and cyclic events in the fish retina, I: HPLC assay of total content, release, and metabolic turnover during different light/dark cycles.. Vis Neurosci.

[15] Douglas RH (1982). The function of photo mechanical movements in the retina of the rainbow trout (*salmo gairdneri*).. J Exp Biol.

[16] Schartau JM, Sjogreen B, Gagnon YL, Kröger RHH (2009). Optical plasticity in the crystalline lenses of the cichlid fish *Aequidens pulcher*.. Curr Biol.

[17] Frail DE, Manelli AM, Witte DG, Lin CW, Steffey ME (1993). Cloning and characterization of a truncated dopamine D1 receptor from goldfish retina: stimulation of cyclic AMP production and calcium mobilization.. Mol Pharmacol.

[18] Cardinaud B, Sugamori KS, Coudouel S, Vincent JD, Niznik HB (1997). Early emergence of three dopamine D1 receptor subtypes in vertebrates. Molecular phylogenetic, pharmacological, and functional criteria defining D1A, D1B, and D1C receptors in European eel *Anguilla anguilla*.. J Biol Chem.

[19] Kanaporis G, Mese G, Valiuniene L, White TW, Brink PR (2008). Gap junction channels exhibit connexin-specific permeability to cyclic nucleotides.. J Gen Physiol.

[20] Goldberg GS, Valiunas V, Brink PR (2004). Selective permeability of gap junction channels.. Biochim Biophys Acta.

[21] Baldridge WH, Vaney DI, Weiler R (1998). The modulation of intercellular coupling in the retina.. Semin Cell Dev Biol.

[22] Voorter CE, Kistler J (1989). cAMP-dependent protein kinase phosphorylates gap junction protein in lens cortex but not in lens nucleus.. Biochim Biophys Acta.

[23] Mathias RT, Kistler J, Donaldson P (2007). The lens circulation.. J Membr Biol.

[24] Vaghefi E, Pontre B, Donaldson PJ, Hunter PJ, Jacobs MD (2009). Visualization of transverse diffusion paths across fiber cells of the ocular lens by small animal MRI.. Physiol Meas.

[25] Lurtz MM, Louis CF (2003). Calmodulin and protein kinase C regulate gap junctional coupling in lens epithelial cells.. Am J Physiol Cell Physiol.

[26] Nguyen TA, Boyle DL, Wagner LM, Shinohara T, Takemoto DJ (2003). LEDGF activation of PKC gamma and gap junction disassembly in lens epithelial cells.. Exp Eye Res.

[27] Churchill GC, Louis CF (1999). Imaging of intracellular calcium stores in single permeabilized lens cells.. Am J Physiol.

[28] Varadaraj K, Kumari S, Shiels A, Mathias RT (2005). Regulation of aquaporin water permeability in the lens.. Invest Ophthalmol Vis Sci.

[29] McCormack CA, McDonnell MT (1994). Circadian regulation of teleost retinal cone movements *in vitro*.. J Gen Physiol.

[30] Malkki PE, Kröger RHH (2005). Visualization of chromatic correction of fish lenses by multiple focal lengths.. J Opt A - Pure Appl Op.

[31] Sroczyński S (1977). Spherical aberration of crystalline lens in the roach *Rutilus rutilus*.. J Comp Physiol A.

